# Probing spatiotemporal PKA activity at the ryanodine receptor and SERCA2a nanodomains in cardomyocytes

**DOI:** 10.1186/s12964-022-00947-8

**Published:** 2022-09-14

**Authors:** Bing Xu, Ying Wang, Sherif M. F. M. Bahriz, Meimi Zhao, Chaoqun Zhu, Yang K. Xiang

**Affiliations:** 1 VA Northern California Health Care System, Mather, CA 95655 USA; 2 Department of Pharmacology, University of California at Davis, Davis, CA 95616 USA; 3 Department of Pharmaceutical Toxicology, China Medical University, Shenyang, 110122 China

**Keywords:** β adrenergic receptor (βAR), β-blockers, Phosphodiesterase (PDE), Ryanodine receptor (RyR), (sarco)endoplasmic reticulum calcium ATPase 2a (SERCA2a), Phospholamban (PLB), Cardiac contractility, Catecholamine

## Abstract

Spatiotemporal regulation of subcellular protein kinase A (PKA) activity for precise substrate phosphorylation is essential for cellular responses to hormonal stimulation. Ryanodine receptor 2 (RyR2) and (sarco)endoplasmic reticulum calcium ATPase 2a (SERCA2a) represent two critical targets of β adrenoceptor (βAR) signaling on the sarcoplasmic reticulum membrane for cardiac excitation and contraction coupling. Using novel biosensors, we show that cardiac β_1_AR signals to both RyR2 and SERCA2a nanodomains in cardiomyocytes from mice, rats, and rabbits, whereas the β_2_AR signaling is restricted from these nanodomains. Phosphodiesterase 4 (PDE4) and PDE3 control the baseline PKA activity and prevent β_2_AR signaling from reaching the RyR2 and SERCA2a nanodomains. Moreover, blocking inhibitory G protein allows β_2_AR signaling to the RyR2 but not the SERCA2a nanodomains. This study provides evidence for the differential roles of inhibitory G protein and PDEs in controlling the adrenergic subtype signaling at the RyR2 and SERCA2a nanodomains in cardiomyocytes.

**Video abstract**

**Supplementary Information:**

The online version contains supplementary material available at 10.1186/s12964-022-00947-8.

## Highlights

- Design a FRET-based biosensor to monitor PKA dynamics at the RyR2 nanodomains in myocytes
- Stimulation of β1AR promotes PKA activity at both RyR2 and SERCA2a nanodomains whereas stimulation of β2AR does not
- Inhibition of PDE3 and PDE4 enhances PKA activity at the baseline and after β2AR stimulation
- Inhibition of Gi selectively permits β2AR signaling to the RyR2 nanodomains but not SERCA2a nanodomains.

**Supplementary Information:**

The online version contains supplementary material available at 10.1186/s12964-022-00947-8.

## Introduction

Cardiac adrenergic stimulation represents the primary regulatory mechanism to enhance cardiac output during stress. Stimulation of βAR promotes PKA activity to enhance cardiac contraction and relaxation in stress response. One of the primary goals of adrenergic stimulation is to enhance calcium cycling by targeting RyR2 and SERCA2a, two ion channels/transporters expressed on the SR membrane [1, 2]. RyR2 resides at the dyadic clefts between the T-tubular membrane and the junctional SR membrane and releases calcium from the SR to the cytoplasm. RyR2 is regulated by local calcium influx via the L-type calcium channel and by PKA phosphorylation of the channel. The increased cytoplasmic concentration of calcium facilitates cross-bridging and filament contraction. In comparison, SERCA2a resides at the distal free SR membrane and is responsible for calcium uptake and cardiac relaxation. SERCA2a is modulated by a negative regulator phospholamban (PLB), which undergoes PKA-mediated phosphorylation and subsequent dissociation of the calcium pump. Notably, a precise regulation of PKA phosphorylation of these substrates is essential to enhance rhythmic heart beating. Moreover, the local cAMP-PKA signaling undergoes alteration in cardiac diseases. For example, the PKA phosphorylation of PLB are usually suppressed in heart failure, whereas the PKA phosphorylation of RyR2 is often elevated in cardiac diseases [3–6]. Until today, there is no direct measurement and comparison of local PKA activity in the RyR2 and SERCA2a nanodomains in cardiac regulation.

Cardiac βARs form local signaling nanodomains based on the distribution of receptors, A kinase anchoring protein (AKAP) scaffold proteins, and downstream signaling and effector components [7, 8]. PKA is anchored on AKAPs to promote local phosphorylation of RyR2 and PLB and increase channel activities [1, 2]. Emerging evidence suggests that RyR2 and SERCA2a represent two distinct signaling nanodomains to conduct adrenergic stimulation of cardiac excitation–contraction coupling. Meanwhile, phosphodiesterases (PDEs) emerge as critical regulators to restrict and fine-tune adrenergic stimulation of cAMP and PKA activity at distinct subcellular nanodomains [9–11]. For example, PDE4D isoforms are shown to associate with both β_1_AR and β_2_AR in cardiac myocytes [12–14]. Stimulation of βARs leads to dynamic dissociation PDE4D isoforms from and recruitment of the PDE4D isoforms to the activated receptors [13, 14]. In addition, cardiac β_2_AR is also known to couple to G_i_, which can restrict the receptor-induced cAMP and PKA activity in the vicinity of the activated receptor [15, 16]. We aim to probe the dynamic PKA activity in RyR2 and SERCA2a nanodomains and the roles of downstream regulatory components in adrenergic subtype dependent PKA activity.

In this study, we designed and applied novel Förster resonance energy transfer (FRET) biosensors based on A-kinase activity reporters (AKARs) [17, 18] to examine local PKA activity at the RyR2 and SERCA2a nanodomains in adult ventricular myocytes (AVMs). Our data revealed that the β_1_AR dominates the adrenergic-induced PKA signaling at the RyR2 and SERCA2a nanodomains in AVMs, whereas stimulation of the β_2_AR leads to minimal PKA activity at these nanodomains. PDE4 and PDE3 control the baseline PKA activity at both nanodomains, whereas phosphatases play a minimal role. Inhibition of PDE3 permits the β_2_AR stimulation of PKA activity at the SERCA2a and RyR2 nanodomains while inhibition of PDE4 preferentially permits the β_2_AR signaling to the SERCA2a nanodomains. In contrast, inhibition of G_i_ only allows the β_2_AR stimulation of PKA activity at the RyR2 but not at the SERCA2a nanodomains. Our data highlight distinct adrenergic subtype signaling regulation at the RyR2 and SERCA2a nanodomains in AVMs.

## Materials and methods

### Animals

Animal studies and experimental protocols were approved by the Institutional Animal Care and Use Committees (IACUC) of the University of California at Davis (protocol number: 20956 and 20,957) and complied with the National Institutes of Health and ARRIVE guidelines. Male Sprague Dawley outbred rats (3–5 months) were used. Male C57BL/6 J mice (2–4 months) were purchased from Jackson Laboratory (Sacramento, CA). Animals were maintained in a standard room with controlled temperature, humidity, and 12–12-h light–dark cycle. Mice and rats were anesthetized with inhalation of 2.0% isoflurane and oxygen before harvesting hearts. All studies are randomized and blinded for data analysis.

### Reagents

Unless specified, all reagents were obtained from Millipore-Sigma (St. Louis, MO). β-adrenergic agonists, isoproterenol (ISO, 100 nmol/L), was applied to cultured myocytes with a β_1_AR antagonist (CGP 20712A, 300 nmol/L) or ISO with a β_2_AR antagonist (ICI 118,551, 100 nmol/L). Inhibitors of PDE2 (EHNA), PDE3 (cilostamide), and PDE4 (rolipram) and Gi (pertussis toxin, PTX) were used as indicated. FKBP-AKAR3 was generated by fusing AKAR3 to the C-terminus of FKBP12.6 into pcDNA3.1, then subcloned into pshuttle vector to generate recombinant padeasy vector for making adenovirus as previously described [17–19]. Adenoviruses containing the FKBP-AKAR3 fusion gene were made and amplified in HEK293 cells. The recombinant viruses were purified with a CsCl_2_ gradient as described [17–19]. We successfully produced the adenovirus with a titer of 10^11^–10^12^ pfu/ml. Adenoviruses expressing cyto-AKAR3 and SR-AKAR3 were described previously [17–19].

### Adult ventricular cardiomyocyte (AVM) isolation from adult mouse, rat, and rabbit

AVMs were isolated as previously described [20–22]. The heart was quickly removed and cannulated to a Langendorf perfusion system. The heart was perfused with the digestion buffer (NaCl 120 mmol/L, NaH2PO4 1.2 mmol/L, KCl 5.4 mmol/L, MgSO4 1.2 mmol/L, NaHCO3 20 mmol/L, Glucose 5.6 mmol/L, Taurine 20 mmol/L, 2,3-Butanedione monoxime 10 mmol/L, PH7.33) and followed with the buffer containing collagenase and protease (pre-digestion solution: 0.05% type II collagenase (Worthington Biochemical, Lakewood, NJ), 0.01% mg type XIV protease (Sigma-Aldrich), and 0.1% BSA; digestion solution: 0.2% type II collagenase, 0.04% type XIV protease, 50 µM CaCl2, and 0.1% BSA). The ventricle was cut and gently titrated into small pieces and further digested with collagenase solution. Isolated AVMs were harvested and recovered in a series of concentration of calcium. Fresh rabbit AVMs were provided by Dr. Donald Bers at University of California at Davis. AVMs were used for acute experiments including western blotting and fractional shortening recording or cultured in serum free M1018 media for FRET assays.

### Western blotting

WT AVMs expressing FKBP-AKAR3 were treated with 100 nmol/L isoproterenol (ISO, Sigma) for 10 min as indicated. The levels of phospho-RyR2 at Ser2807 (pRyRS2807) and Ser2814 (pRyRS2814), RyR2, phospho-PKA substrate (RRXS*/T*) (pPKAsub), and FKBP-AKAR3 were detected in western blots. The treated AVMs from indicated mice were lysed with RIPA buffer supplement with proteinase and phosphatase inhibitors. Immunoblotting was applied to detect the expression of pRyR2-S2807 (ab59225, Abcam, Cambridge, MA), pRyR2-S2814 (A010-31, Badrilla, England), RyR2 (MA3-925, Thermofisher, IL), pPKAsub (9624, Cell Signaling, Danvers, MA), FKBP-AKAR3 (GFP, 632592, Clontech, CA), and γ-tubulin (T6557, Sigma-Aldrich, St Louis, MO). IRDye 680RD goat anti-rabbit IgG secondary antibody (926–68071, LI-COR, Lincoln, NE) and IRDye 800CW goat anti-mouse IgG secondary antibody (926–32210, LI-COR, Lincoln, NE) were used for multi-color detection. PVDF membranes were scanned on Biorad Chemdoc MP imaging systems (Biorad, Hercules, CA). The optical density of the bands was analyzed with NIH Image J software (https://imagej.nih.gov/ij/).

### Fluorescence resonance energy transfer (FRET) assay

FRET assay was carried out following the method reported before [17–19, 22]. Briefly, AVMs were cultured on laminin-coated coverslips in serum-free M1018 media (PH 7.35, Sigma) supplement with 6.25 μmol/L blebbistatin and infected with FKBP-AKAR3 or SR-AKAR3 biosensors at a MOI of 100 for 36 h [17, 18] before recording on a Leica inverted fluorescence microscope (DMI3000 B, Buffalo Grove, IL). Myocytes were recorded using Metafluor software (Molecular Devices, Sunnyvale, CA). Cyan fluorescent protein (CFP) and yellow fluorescent protein (YFP) were imaged by filter 475DF40 and filter 535DF25 every 20 s, with an exposure time of 200–500 ms. After recording the baseline, AVMs were treated with ISO (10 pmol/L, 100 pmol/L, 1 nmol/L, 10 nmol/L, 100 nmol/L, and 1 μmol/L), 10 min ICI 118,551 (I127, Sigma-Aldrich), CGP 20712a (C125, Sigma-Aldrich), EHNA (324,630, Calbiochem), cilostamide (Cilo, 0915, Tocris Bioscience), rolipram (Roli, R6520, Sigma-Aldrich), and PTX (300 ng/ml, 3 h) as indicated. Fluorescence emission intensity at 545 nM (YFP) and 480 nM (CFP) was subjected to background subtraction. YFP/CFP ratio was analyzed as F/F0, in which F is at time t and F0 is the baseline. An increase in the YFP/CFP indicates the activation of PKA. The numbers of cells were labeled in the figures.

### AVM contractility

As previously reported [20, 21, 23], AVMs were placed on a dish and paced at 1 Hz with a SD9 stimulator (Grass Technology, Warwick, RI). Metamorph software was used to image beating cells in a bright field before and 5 min after drug administration with a Zeiss AX10 inverted fluorescence microscope (Zeiss AX10, Dublin, CA). Fractional shortening was analyzed in movies acquired in bright field using Metamorph software [20, 21, 23]. The numbers of cells were labeled in the figures.

### Statistical analysis

Pooled data were represented as the mean ± SEM. Male animals were used for all experiments. Fully blinded analysis was performed with different persons carrying out the experiments and analysis, respectively. All data were included for the analysis. Representative figures/images reflected the average levels of the experiments. Normality of the data was assessed using the Shapiro–Wilk test in GraphPad Prism 9 with significance at alpha = 0.05 (GraphPad Inc., San Diego, CA). If N <  = 6, the data were assumed normality due to the central limit theorem. Comparisons between two groups were performed by paired and unpaired Student’s *t*-test and by nested Student’s *t*-test. Comparisons between more than two groups were performed by one-way (nested) ANOVA or two-way (nested) ANOVA followed by Tukey’s post-hoc using Prism 9.0 software (GraphPad). A value of two-tailed *P* < 0.05 was considered statistically significant.

## Results

**Fig. 1:**
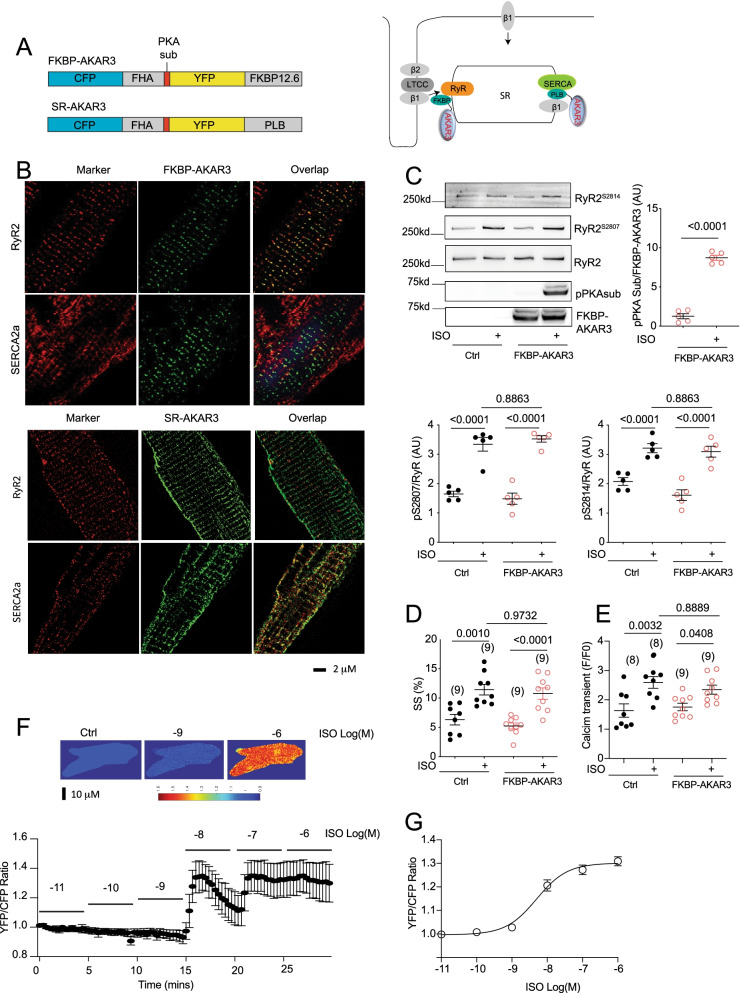
Development of biosensors to detect local PKA activity in AVMs. **A** Schematics of genetically encoded FRET-based FKBP-AKAR3 and SR-AKAR3 biosensors and the subcellular distribution in AVMs. **B** Confocal images show FKBP-AKAR3 and SR-AKAR3 colocalize with RyR2 and  SERCA2a, respectively, in rabbit AVMs. **C** Western blot shows PKA phosphorylation of FKBP-AKAR3 and endogenous RyR2 expressed in rabbit AVMs after stimulation with 100 nmol/L of ISO. The phosphorylation of RyR2 at serine 2807 and 2814 and FKBP-AKAR3 at the PKA substrate site were detected with specific antibodies and quantified in dot plots (N = 5). **D** and **E** AVMs with and without expressing FKBP-AKAR3 were stimulated with 100 nmol/L of ISO, sarcomere shortening (SS) and calcium transient of rabbit AVMs were measured before and after ISO stimulation. The maximal increases in SS and calcium transient are summarized in dot plots. Data represent mean ± SEM of indicated number of AVMs from rabbits. **F** Images show the YFP/CFP ratio in rabbit AVMs before and after stimulation with different concentrations of ISO, the representative curve shows the time course of YFP/CFP ratio after stimulation. **G** A dose response curve of the maximal increases in YFP/CFP ratio after stimulation with different concentrations of ISO. Data represent mean ± SEM of AVMs from mice (EC50, 5.0 * 10^–9^ mol/L). p values were obtained by Student *t*-test between two groups or by one-way ANOVA analysis followed with Tukey’s multiple comparison test. AU, arbitrary unit

To examine local PKA activity at the RyR2 nanodomains, we generated a new PKA FRET biosensor anchored to the RyR2 complex by fusing AKAR3 to FKBP12.6 [24], an auxiliary protein binding to RyR2, which has been successfully used to anchor calcium and cAMP biosensors previously [25, 26] (Fig. 1A). The FKBP-AKAR3 biosensor colocalized with RyR2 but not with SERCA2a in mouse AVMs (Fig. 1B). In comparison, an AKAR3 anchored to SERCA2a complex (SR-AKAR3) [17, 18] colocalized with SERCA2a but not RyR2 (Fig. 1B). FKBP-AKAR3 biosensor expressed rabbit AVMs displayed robust increases in phosphorylation at the PKA site after adrenergic stimulation with isoproterenol (ISO) (Fig. 1C and D). The expression of FKBP-AKAR3 did not affect adrenergic stimulation of PKA phosphorylation of RyR2 at serine 2807 and  phosphorylation  at serine 2814 (Fig. 1C and D and Additional File 1). The expression of FKBP-AKAR3 did not affect adrenergic stimulation of calcium transient and sarcomere shortening responses (Fig. 1E and F). Moreover, the FKBP-AKAR3 biosensor displayed a dose-dependent increase in FRET ratio in response to ISO stimulation (EC50, 5.0 ×X 10^–9^ M, Fig. 1G and H). These data indicate that the FKBP-AKAR3 has appropriate targeting to image PKA dynamics at the RyR2 nanodomains in AVMs without affecting endogenous adrenergic regulation of PKA phosphorylation and contractile function.

**Fig. 2:**
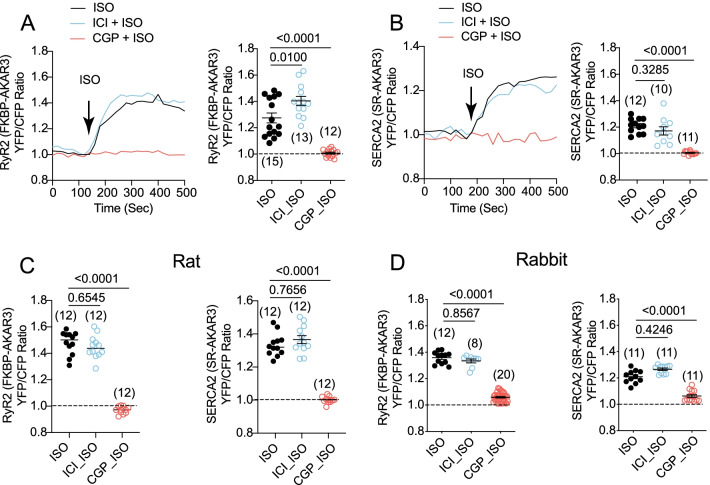
Biosensors detect distinct local adrenergic signaling at the RyR2 and SERCA2 nanodomains in mouse, rat, and rabbit AVMs. **A** and **B** FKBP-AKAR3 and SR-AKAR3 biosensors were expressed in mouse AVMs. Time courses show changes in YFP/CFP ratio of FKBP-AKAR3 and SR-AKAR3 in AVMs after stimulation with ISO (100 nmol/L) in the presence of β_1_AR antagonist CGP20712a (CGP, 300 nmol/L) and β_2_AR antagonist ICI118551 (ICI, 100 nmol/L). The maximal changes in FRET ratio after drug treatment were plotted. Data represent mean ± SEM of indicated number of AVMs from rats. **C** The maximal increases in YFP/CFP ratio of FKBP-AKAR3 and SR-AKAR3 in rat AVMs after stimulation with ISO (100 nmol/L) or in the presence of β_1_AR antagonist CGP (300 nmol/L) or β_2_AR antagonist ICI (100 nmol/L). Data represent mean ± SEM of indicated number of AVMs from rats. **D** The maximal increases in YFP/CFP ratio of FKBP-AKAR3 and SR-AKAR3 in rabbit AVMs after stimulation with ISO (100 nmol/L) or in the presence of CGP or ICI. Data represent mean ± SEM of indicated number of AVMs from rabbits. p values were obtained after one-way ANOVA analysis followed by Tukey’s test

We then applied FKBP-AKAR3 and SR-AKAR3 to analyze the adrenergic induced-PKA signaling dynamics within these two distinct local SR nanodomains in AVMs. Stimulation of cardiac βAR with ISO induced robust increases in AKAR3 FRET ratio at the RyR2 and SERCA2a nanodomains in mouse AVMs (Fig. 2A and B). Moreover, inhibiting β_1_AR rather than β_2_AR abolished the PKA FRET responses at both nanodomains (Fig. 2A and B). Similar data were observed in rat and rabbit AVMs, indicating that adrenergic signaling to the RyR2 and SERCA2a nanodomains is conserved among these species (Fig. 2C and D). These data suggest that the β_1_AR is the dominant cardiac βAR subtype to promote PKA activity at the RyR2 and SERCA2a nanodomains.

**Fig. 3:**
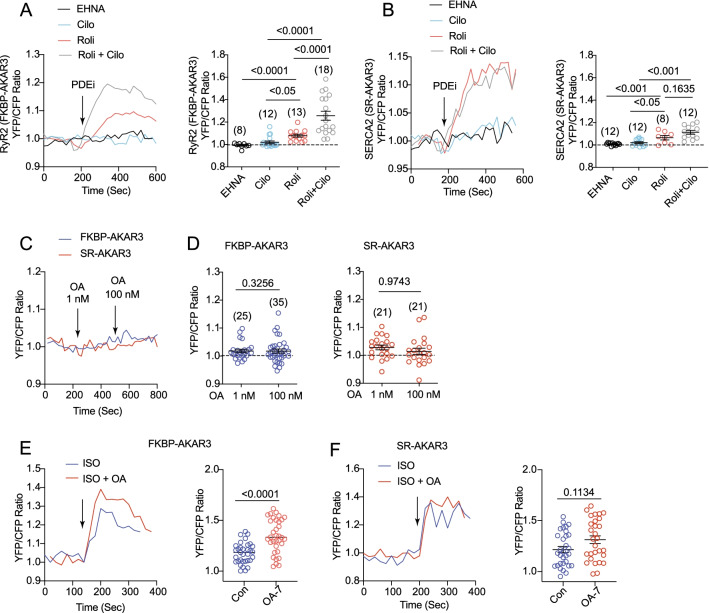
Phosphodiesterases and phosphatases control the baseline PKA activity at the RyR2 and SERCA2 nanodomains in AVMs. Rat AVMs expressing FKBP-AKAR3 and SR-AKAR3 were treated with drugs as indicated**. A** and **B** Time courses show the changes in YFP/CFP ratio before and after addition of inhibitor of PDE2 *erythro*-9-(2-Hydroxy-3-nonyl) adenine hydrochloride (EHNA 10 µmol/L), PDE3 (cilostamide, Clio, 1 µmol/L), PDE4 (rolipram, Roli, 10 µmol/L), or both Cilo and Roli. The maximal increases in YFP/CFP ratio before and after drug treatment were plotted. **C** and **D** Time courses show the changes in YFP/CFP ratio before and after addition of phosphatase inhibitor (1 nmol/L and 100 nmol/L, okadaic acid, OA) as indicated. The maximal increases in YFP/CFP ratio of FKBP-AKAR3 and SR-AKAR3 in AVMs after drug treatment were plotted. **E** and **F** co-treated with ISO (10 nmol/L) together with phosphatase inhibitor OA (100 nmol/L) as indicated. Time courses show the changes in YFP/CFP ratio before and after addition of drugs. The maximal increases in YFP/CFP ratio of FKBP-AKAR3 and SR-AKAR3 in AVMs before and after stimulation were plotted. Dot plots represent mean ± SEM of indicated number of AVMs from mice. p values were obtained by Student *t*-test between two groups or by one-way ANOVA analysis followed with Tukey’s multiple comparison test

PDEs and phosphatases are critical regulators of local cAMP and PKA activity in AVMs [7–11]. PDE3 and PDE4 are shown to associated with RyR2 and SERCA2a complexes in myocytes [5, 27, 28], which can influence the baseline PKA activity in these nanodomains. We analyzed the contribution of cardiac PDE families in controlling local PKA activity at the RyR2 and SERCA2a nanodomains in AVMs. Neither PDE2 nor PDE3 inhibition affected the PKA activity at the RyR2 and SERCA2a nanodomains (Fig. 3A and B). In comparison, inhibiting PDE4 promoted an increase in PKA activity at the RyR2 and SERCA2a nanodomains (Fig. 3A and B). Moreover, inhibition of PDE3 and PDE4 together synergistically promoted increases in PKA activity at the RyR2 nanodomains relative to individual inhibitors. In comparison, PDE3 and PDE4 double inhibition did not further enhance the PKA activity at the SERCA2a nanodomains relative to PDE4 inhibitor alone (Fig. 3A and B). These data indicate that PDE4 is the predominant PDE family maintaining the baseline PKA activity at the RyR2 and SERCA2a nanodomains in the heart. PDE3 plays an additional role in managing PKA activity at the RyR2 nanodomains. Meanwhile, inhibition of phosphatase 1 and phosphatase 2A with okadaic acid (1 nM and 100 nM, respectively) did not affect the baseline PKA activity at the RyR2 or SERCA2a nanodomains (Fig. 3C and D). Inhibition of phosphatases, however, enhanced adrenergic (ISO)-induced PKA activity at the RyR2 nanodomains (Fig. 3E and F).

**Fig. 4:**
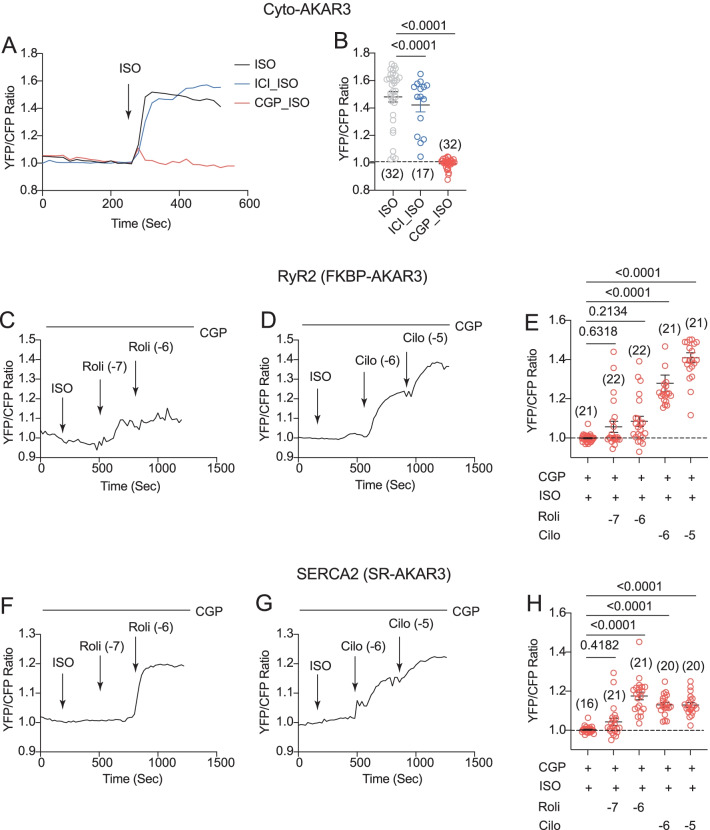
Phosphodiesterases restrict β_2_AR signaling at the RyR2 and SERCA2 nanodomains in AVMs. Rat AVMs expressing Cyto-AKAR3, FKBP-AKAR3, and SR-AKAR3 were treated with drugs as indicated**. A** and **B** Time courses show changes in YFP/CFP ratio of Cyto-AKAR3 in rat AVMs induced by ISO (100 nmol/L) after 5 min pretreatment of β_1_AR antagonist CGP (300 nmol/L) and β_2_AR antagonist ICI (100 nmol/L). The maximal changes in FRET ratio after drug treatment were plotted. **C–E** Time courses show changes in YFP/CFP ratio of FKBP-AKAR3 induced by ISO (100 nmol/L) after 5 min pretreatment with β_1_AR antagonist CGP (300 nmol/L), which was then followed with addition of inhibitor of PDE4 (Roli, 100 nmol/L and 1 µmol/L) and PDE3 (Cilo, 1 and 10 µmol/L) as indicated. The maximal changes in FRET ratio after drug treatment were plotted. **F–H** Time courses show changes in YFP/CFP ratio of SR-AKAR3 induced by ISO (100 nmol/L) after 5 min pretreatment with β_1_AR antagonist CGP (300 nmol/L), which was then followed with addition of inhibitor of PDE4 (Roli, 100 nmol and 1 µmol/L) and PDE3 (Cilo, 1 and 10 µmol/L) as indicated. The maximal changes in FRET ratio after drug treatment were plotted. Dot plots represent mean ± SEM of indicated number of AVMs from rats. p values were obtained by one-way ANOVA analysis followed with Tukey’s multiple comparison test

The lack of β_2_AR-induced PKA activity at the RyR2 and SERCA2a nanodomains indicates that the downstream signaling components such as PDE may restrict the receptor signaling. We then detected PKA activity in the bulky cytoplasm. Stimulation of cardiac βAR with ISO induced robust increases in FRET ratio of cyto-AKAR3 at the cytoplasm in AVMs (Fig. 4A and B). Surprisingly, inhibiting β_1_AR almost abolished the ISO induced PKA FRET responses, whereas inhibiting β_2_AR did not affect ISO-induced PKA activity (Fig. 4A and B). These data indicate that the β_2_AR-induced PKA activity is restricted in the vicinity of the activated receptor. After stimulation of β_2_AR with ISO in the presence of β_1_AR blocker CGP20712a, inhibition of PDE3 but not PDE4 significantly potentiated β_2_AR-induced PKA activity at the RyR2 nanodomains (Fig. 4C–E). Inhibition of PDE3 or PDE4 significantly potentiated β_2_AR-induced PKA activity at the SERCA2a nanodomains (Fig. 4F–H).

**Fig. 5:**
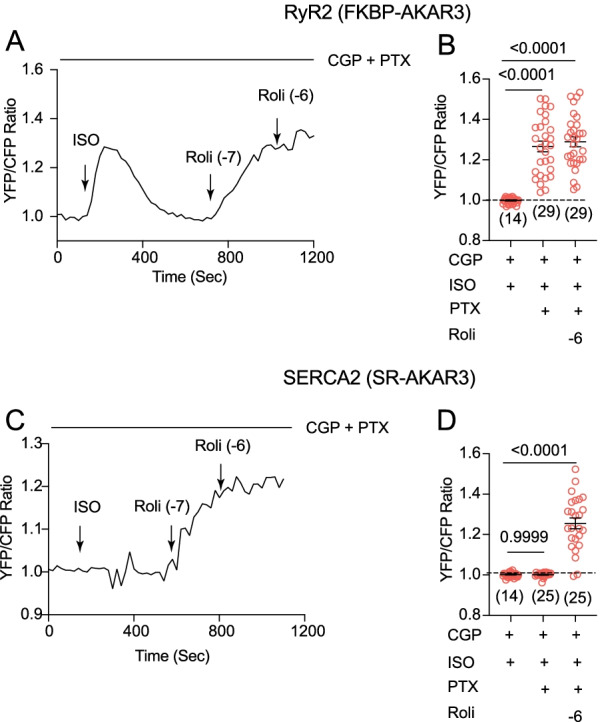
Inhibitory G protein selectively restricts β_2_AR signaling at the RyR2 nanodomains in AVMs. Rat AVMs expressing FKBP-AKAR3 and SR-AKAR3 were treated with drugs as indicated**. A** and **B** Time courses show changes in YFP/CFP ratio of FKBP-AKAR3 in AVMs after drug treatments. AVMs were pretreated with G_i_ inhibitor pertussis toxin (PTX, 300 ng/ml, 3 h) and β_1_AR antagonist CGP (300 nmol/L, 10 min) before stimulation with ISO (100 nmol/L), and followed with inhibitor of PDE4 (Roli, 100 nmol and 1 µmol/L) as indicated. The maximal changes in FRET ratio after drug treatment were plotted. **C** and **D** Time courses show changes in YFP/CFP ratio of SR-AKAR3 in AVMs after drug treatments. AVMs were pretreated with G_i_ inhibitor PTX (300 ng/ml, 3 h) and β_1_AR antagonist CGP (300 nmol/L, 10 min) before stimulation with ISO (100 nmol/L), and followed with inhibitor of PDE4 (rolipram, 100 nmol and 1 µmol/L) as indicated. The maximal changes in FRET ratio after drug treatment were plotted. Dot plots represent mean ± SEM of indicated number of AVMs from rats. *p* values were obtained by one-way ANOVA analysis followed with Tukey’s multiple comparison test

Meanwhile, the β_2_AR is known to couple to G_i_ to restrict cAMP-PKA signaling in cardiac myocytes [15, 16]. After stimulation of β_2_AR with ISO in the presence of β_1_AR blocker CGP20712a, inhibition of G_i_ with pertussis toxin enhanced a transient PKA activity at the RyR2 nanodomains, and the addition of PDE4 inhibitor promoted a sustained PKA activity (Fig. 5A and B). In comparison, inhibition of G_i_ with pertussis toxin did not produce PKA activity at the SERCA2a nanodomains, whereas additional inhibition of PDE4 recovered PKA activity at the SERCA2a nanodomains (Fig. 5C and D). These data indicate that G_i_ and PDE4 differentially restrict the β_2_AR-induced PKA activity at the RyR2 and SERCA2a nanodomains on the SR membrane.

## Discussion

RyR2 and SERCA2a-mediated SR calcium release and uptake critically affect myocyte calcium cycling, thus regulating cardiac systolic and diastolic function [1, 2]. In this study, utilizing the new generated biosensors, we revealed distinct subcellular adrenergic signaling at the RyR2 and SERCA2a nanodomains in AVMs from mice, rats, and rabbits. Our data show that cardiac β_1_AR is the dominant subtype to promote PKA signaling at the RyR2 and SERCA2a nanodomains, whereas cardiac β_2_AR minimally enhances local PKA activity in these nanodomains. Additional studies show that PDE3 and PDE4 are critical in restricting cardiac β_2_AR signaling to these SR membrane nanodomains. Moreover, G_i_ plays an essential role for limiting cardiac β_2_AR signaling to the RyR2 but blocking G_i_ does not allow β_2_AR signaling to the SERCA2a nanodomains. Our data uncover a differential role of G_i_ and PDEs in restricting local PKA activity at the RyR2 and SERCA2a nanodomains during adrenergic subtype stimulation (Fig. 5E).

β-adrenergic signaling increases RyR2 and SERCA2a function by PKA-dependent phosphorylation of RyR2 and PLB, the negative regulator of SERCA2a. The opening RyR2 increases intracellular calcium concentration for enhancing myofilament contraction, thus playing a significant role in systolic function. Among PDE genes expressed in rodent hearts, PDE4 and PDE3 represent the major PDE enzymes that are responsible for cAMP degradation [29]. Our data show that PDEs but not phosphatases are critical in maintaining baseline PKA activity at RyR2 and SERCA2a nanodomains in AVMs. Notably, PDE4 is the dominant player in maintaining baseline PKA activity in RyR2 and SERCA2a nanodomains, consistent with the previous report that PDE4D isoforms are identified in RyR2 and SERCA2a complexes [5, 30]. However, inhibition of PDE3 can further enhance local PKA activity at the RyR2 nanodomains, indicating that PDE3 controls the baseline RyR2 activity, consistent with the inotropic effects of PDE3 inhibitors in humans and rodents [10]. Our data show a minimal role of PDE2 in regulating local PKA activity in both RyR2 and SERCA2a nanodomains. Nevertheless, the functions of these enzymes may be changed in diseased states, in which PDE2 and PDE3 can be more significant in controlling local PKA activity at the RyR2 while the role of PDE4 is diminished due to dissociation from RyR2 complex [5, 26, 31].

The β_1_AR is known to be distributed to the T-tubular membrane, which may be close to the dyad containing the RyR2 nanodomains on the SR membrane. The β_1_AR induces robust PKA activity at the RyR2 nanodomains, supporting the proximity of the receptor to the RyR2 machinery for tight regulation of calcium cycling during adrenergic stimulation. Notably, the cardiac β_1_AR undergoes desensitization and degradation in heart failure [32, 33]. Further study will help understand how the cardiac β_1_AR is uncoupled from the RyR2 nanodomains during the development of cardiac diseases. Meanwhile, we have recently characterized an internal pool of β_1_AR associated with SERCA2a on the SR [34]. In agreement, our observations show that the β_1_AR is also the dominant subtype to stimulate local PKA activity at the SERCA2a nanodomains. These data support the notion that the distinct pools of cardiac β_1_AR promote local cAMP-PKA activity in the critical signaling nanodomains to precisely regulate ion channel activity for cardiac contraction response.

The cardiac β_2_AR displays a much more restricted action in AVMs [35]. While previous studies rule out the possible internal distribution of the β_2_AR in AVMs [34, 36], the receptor has been detected in the T-tubular membrane [37], thus potentially accessing the RyR2 nanodomains. However, our data show that stimulation of β_2_AR minimally enhances PKA activity at the RyR2 and SERCA2a nanodomains as well as in the bulky cytoplasm, in contrast to those induced by β_1_AR. Further analysis reveals that both G_i_ and PDEs restrict the β_2_AR signaling. Inhibition of G_i_ is enough to enhance the β_2_AR-induced PKA activity at the RyR2 nanodomains but did not enhance PKA activity at the SERCA2a nanodomains, indicating the removal of G_i_ only permits a transient β_2_AR signaling near the T-tubule and dyad. In comparison, inhibition of PDE3 and PDE4 is sufficient to enhance the β_2_AR-induced PKA activity at the RyR2 and SERCA2a nanodomains. The dynamic receptor association and dissociation of PDE4D isoforms after agonist stimulation can also contribute to the differential regulation of βAR subtype-specific signaling in AVMs. While stimulation of the β_1_AR promotes dissociation of PDE4D8 from the activated receptor [13], stimulation of the β_2_AR leads to dissociation of PDE4D9, a transient dissociation of PDE4D8, and a recruitment of PDE4D5 [14]. Thus, the PDE-free β_1_AR may send signal to the distance, whereas the PDE-bound β_2_AR has a local signal in the receptor vicinity for phosphorylation of the receptor and calcium channel in the complex [38, 39]. Together, these data suggest that while G_i_ and PDEs restrict cAMP-PKA activity in the vicinity of the activated receptor, inhibition of G_i_ only permits limited regional diffusion, which is also transient due to PDE-mediated cAMP hydrolysis. In contrast, additional inhibition of PDE associated with RyR2 and SERCA2a is necessary for the β_2_AR signaling to the ion channels and transporters in hearts. Thus the β_2_AR signaling is more restricted in AVMs relative to those in neonatal cardiac myocytes with less developed T-tubular structure [19]. Meanwhile, previous studies show that stimulation of β_2_AR with clenbuterol can induce a small signal in the cytoplasm [40], the observed effects may be due to a partial activation of the β_1_AR at the concentration. While our study was performed on rodents and rabbits, the β_2_AR subtype accounts for a low percentage of total βARs in human hearts [41]. Therefore, the specie-dependent difference should be considered when extrapolating the findings to human. Given the increased role of the β_2_AR in heart failure, it is essential to understand the alternation of these local signaling in diseased hearts in future studies.

Multiple strategies have been successfully deployed to target genetically encoded biosensors to detect local signaling in subcellular nanodomains in AVMs. These include using regulatory proteins such as phospholamban, troponin T, FKBP, and A kinase-anchor proteins, and structural and scaffold proteins such as junctophilin as anchors [17, 22, 25, 26, 42, 43]. All these targeting strategies have advantages and disadvantages in probing local signaling nanodomains. The limitation of the current study is that FKBP can only target a pool of RyR2 [24] and FKPB can potentially dissociate from the RyR2 complexes [44] under chronic stimulation and pathological conditions, which may affect the readout. In a biological paradigm, one should corroborate the signaling detection by biosensors with other biochemical and functional evidence. Nevertheless, these targeted biosensors have greatly enhanced our understanding local signaling remodeling in both physiological and pathological conditions [17, 22, 25, 26, 42, 43].

In summary, we have detected dynamic PKA activity induced by adrenergic subtypes at the RyR2 and SERCA2a nanodomains in AVMs from three species. Our study reveals the differential roles of G_i_ and PDEs in controlling local PKA signaling induced by β_2_AR, which will help us understand the role of these signaling regulations in physiological and pathological conditions.

## Supplementary Information

**Additional file 1**. Figure 1C Original Gels.

## Data Availability

The datasets supporting the conclusions of this article are included within the article and its Additional files.

## Abbreviations

AR: Adrenoceptors
AVM: Adult ventricular cardiomyocyte
AKAR: A kinase activity reporter
FRET: Förster resonance energy transfer
FS: Fractional shortening
ISO: Isoproterenol
PDE4D: Phosphodiesterase 4D
PLB: Phospholamban
PM: Plasma membrane
PKA: Protein kinase A
RyR2: Ryanodine receptor 2
SS: Sarcomere shortening
SERCA2: (Sarco)endoplasmic endoplasmic reticulum Ca^2+^^−^ATPase 2
SR: Sarcoplasmic reticulum
WT: Wild type
cAMP: Cyclic AMP
