# Supplementary figures and images for: Probing spatiotemporal PKA activity at the ryanodine receptor and SERCA2a nanodomains in cardomyocytes
Source: Cell Commun Signal. 2022 Sep 14;20:143. doi: 10.1186/s12964-022-00947-8 (PMC9472443; doi:10.1186/s12964-022-00947-8)

Figure 1C Original Gels

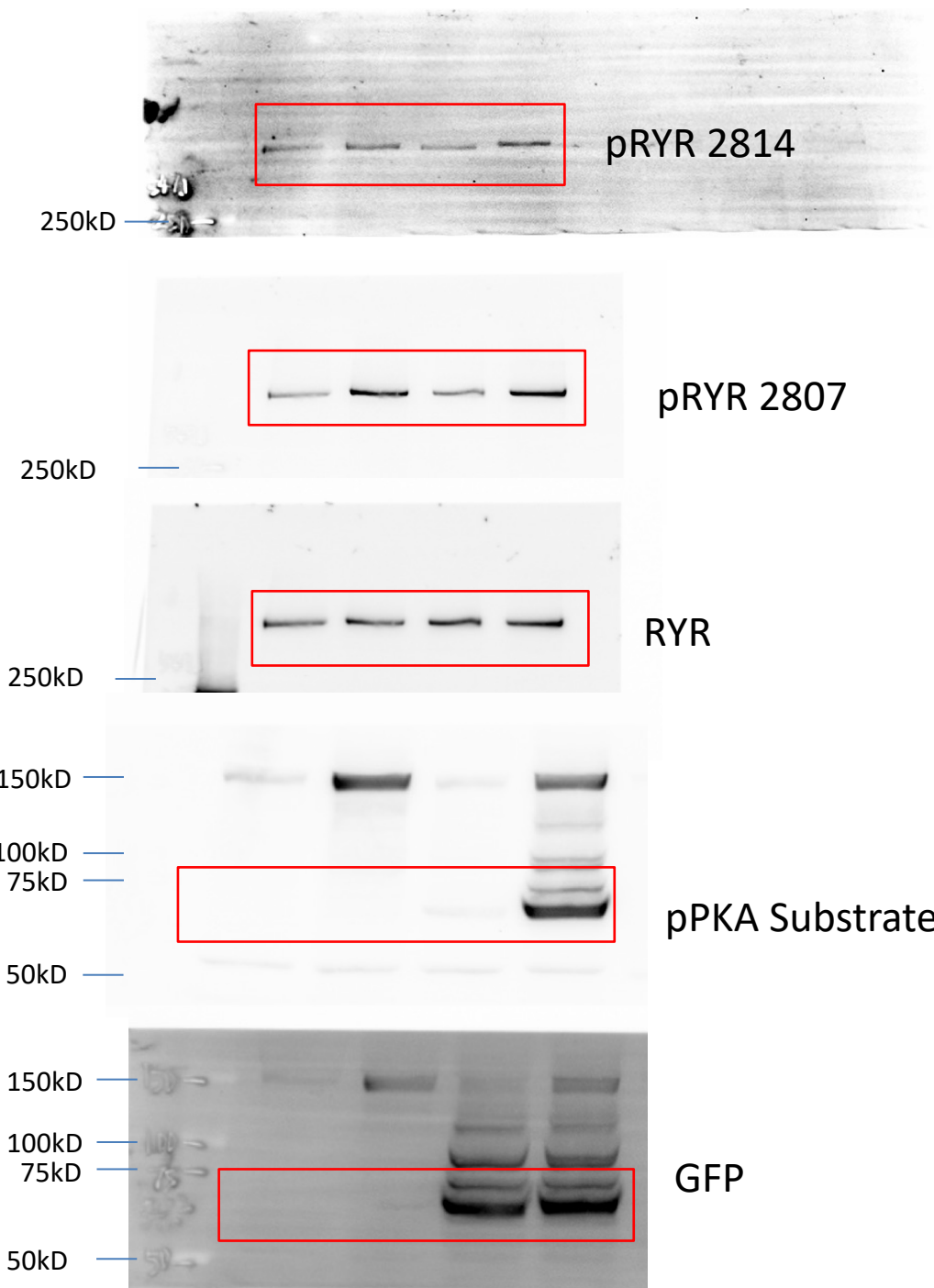

Supplement: Supplementary file 2 — Additional file 1. Figure 1C Original Gels. [file 12964_2022_947_MOESM2_ESM.pdf]
